# *Astragalus* Polysaccharide Improves Insulin Sensitivity via AMPK Activation in 3T3-L1 Adipocytes

**DOI:** 10.3390/molecules23102711

**Published:** 2018-10-21

**Authors:** Ruixin Zhang, Xuze Qin, Ting Zhang, Qian Li, Jianxin Zhang, Junxing Zhao

**Affiliations:** Department of Animal Sciences and Veterinary medicine, Shanxi Agricultural University, Taigu 030801, China; ruixinzhang1719@163.com (R.Z.); XuZeQin799@gmail.com (X.Q.); tzh3058@163.com (T.Z.); qianli2418@163.com (Q.L.); sxndzjx@163.com (J.Z.)

**Keywords:** *astragalus* polysaccharide, adipogenesis, proliferation, insulin sensitivity, AMPK

## Abstract

*Astragalus* polysaccharide (APS) is an important bioactive component of *Astragalus membranaceus* which is used as an anti-diabetes herb in traditional Chinese medicine. The objective of this study was to investigate the effects and mechanisms of APS on insulin-sensitizing of adipocytes. Mouse 3T3-L1 preadipocytes were used as a model. The results showed that APS increased preadipocytes proliferation in a dose dependent manner, and 0.1 μg/mL APS sufficiently increased Proliferating Cell Nuclear Antigen (PCNA) content (*p* < 0.01). Moreover, APS enhanced intracellular lipid accumulation and mRNA expression of proliferator-activated receptor γ (PPARγ, *p* < 0.01), CCAAT/enhancer binding protein α (C/EBPα, *p* < 0.01) and fatty acid binding protein (*a*P2, *p* < 0.01). As expected, corresponding protein contents were elevated. Importantly, APS increased 2-(*N*-(7-Nitrobenz-2-oxa-1,3-diazol-4-yl)Amino)-2-Deoxyglucose (2-NBDG) uptake (*p* < 0.01). Meanwhile, both mRNA and protein content of glucose transporter 4 (Glut4) were elevated by APS (*p* < 0.01). The APS treatment enhanced tyrosine phosphorylation of insulin receptor substrate 1 (IRS1, *p* < 0.05) and phosphor-Akt content (*p* < 0.01). Besides, phosphorylated AMP-activated protein kinase (AMPK) content was increased in the APS treated cells (*p* < 0.01). Taken together, APS improved insulin sensitivity by enhancing glucose uptake, possibly through AMPK activation. These results suggested that APS might be a therapeutic candidate for insulin resistance.

## 1. Introduction

Type 2 diabetes mellitus (T2DM) is a chronic metabolic disorder, and its prevalence has been increasing steadily worldwide [1]. T2DM patients have a lower insulin utilization ability in metabolic organs and tissues, leading to high blood glucose levels and other complications, such as obesity, hypertension, atherosclerosis, liver failure, and certain cancers [2]. Insulin resistance (IR) refers to the reduced biological efficacy of insulin on effector organs. IR and the consequences of declined glucose uptake and elimination in surrounding tissues, including the liver, skeletal muscle, and adipose tissues, are the main contributors of the T2DM pathogenesis [3]. Thus, therapeutic agents that improve the insulin sensitivity have received considerable attention.

AMP-activated protein kinase (AMPK) is recognized as a critical regulator of energy metabolism [4], and its activity is positively correlated with insulin sensitivity in different tissues and organs [5,6]. Several insulin sensitizing agents targeted at AMPK activation have been developed to improve hyperglycemia [7]. In adipocytes, the glucose transporter 4 (Glut4) mediated glucose uptake in response to insulin is attributed to Akt protein activity. The binding of insulin to the its receptor induces phosphorylation of insulin receptor substrates 1 (IRS1) and subsequent activation of phosphoinositide 3-kinase (PI3K), which further leads to activation of Akt, inducing Glut4 translocation from intracellular storage compartments to the plasma membrane [8].

Traditional Chinese medicine (TCM) has been developed over thousands of years, and it is becoming widely recognized throughout the world because of its potential therapy for a number of diseases and physiological conditions and as an abundant source for new drug discovery [9]. The dry roots of *Astragalus membranaceus* have long been used as an important component of herbal prescriptions in TCM [10]. *Astragalus* polysaccharide (APS) is the major active component of the water extract of *Astragalus* roots. Previous studies demonstrated the diversity of potential effects of APS on antioxidation, anti-hypertensive, anti-tumor, and immunomodulatory, etc. [11]. Importantly, APS alleviates diabetes and diabetes related complications. In a T2DM animal model, APS can effectively attenuate insulin resistance in both muscle and liver [12,13], but its regulatory effects and mechanisms on adipose tissue remain unclear.

Adipose tissue is one of the major targets of insulin, and disruptions in glucose uptake in adipose tissue is associated with insulin resistance [14]. Both proliferator-activated receptor γ (PPARγ) and CCAAT/enhancer binding protein α (C/EBPα) are key regulators of adipogenesis, while the activation of PPARγ has been suggested to improve insulin sensitivity at a different step of the insulin signaling pathway [15]. Mouse 3T3-L1 pre-adipocytes are widely used in examining insulin sensitizing activity of anti-diabetic compounds. In the present study, a direct role of APS on the proliferation and adipogenesis of 3T3-L1 preadipocytes was investigated. Moreover, we explored whether APS could effectively improve insulin sensitivity and whether the AMPK signaling pathway was involved. 

## 2. Results

### 2.1. APS Promoted 3T3-L1 Pre-Adipocytes Proliferation

**Figure 1:**
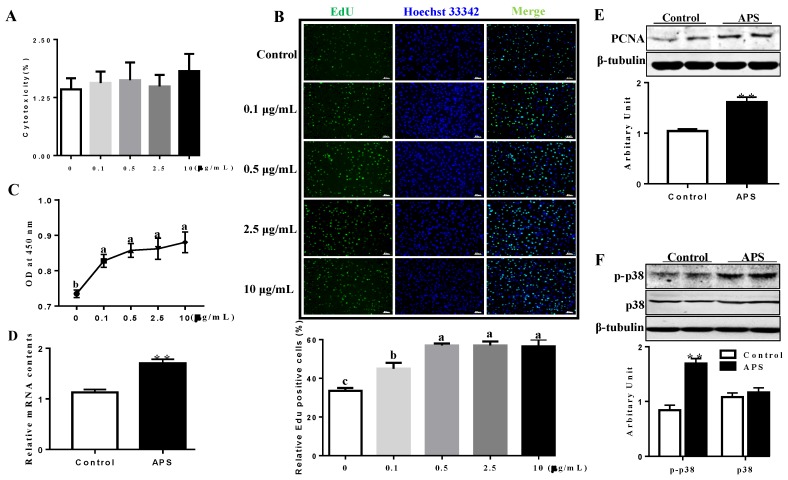
*Astragalus* polysaccharide (APS) promoted 3T3-L1 pre-adipocytes proliferation. (**A**) Cellular cytotoxicity under different concentration of APS was determined by lactate dehydrogenase (LDH) assay. (**B**) EdU staining, scar bar stands for 100 μm. (**C**) Cell viability determined by Cell Counting Kit-8 (CCK8) assay. (**D**) mRNA expression of Proliferating Cell Nuclear Antigen (PCNA). (**E**) PCNA protein content. (**F**) p-p38 and p38 protein content. n = 6, ** *p* < 0.01. Different letters mean significant difference.

The effects of APS on cellular cytotoxicity were determined by a Lactate dehydrogenase (LDH) assay. As shown in Figure 1, no cytotoxicity was observed at certain concentrations of APS (from 0.1 to 10 μg/mL) (Figure 1A). AMPK was activated between 6–12 h after APS administration (Supplementary Figure A). Activation of AMPK by AICAR inhibited 3T3-L1 preadipocytes proliferative ability, and meanwhile, addition of APS increased EdU positive staining cell numbers (Figure 1B), and this effect was obviously enhanced when AMPK activity was blocked by Compound C (Supplementary Figure B). Accordingly, Cell Counting Kit-8 (CCK-8) assay showed that APS promoted 3T3-L1 pre-adipocytes proliferation in a dose dependent manner (Figure 1C). Compared with control cells, addition of 0.1 μg/mL APS sufficiently increased Proliferating Cell Nuclear Antigen (PCNA) mRNA expression (*p* < 0.01, Figure 1D). As expected, PCNA protein content was increased when cells were treated with APS (*p* < 0.01, Figure 1E). Although p38 protein content was not affected by APS, an increased p-p38 protein level was observed in APS treated cells (*p* < 0.01, Figure 1F). 

### 2.2. Effect of APS on Adipogenesis in 3T3-L1 Pre-Adipocytes

**Figure 2:**
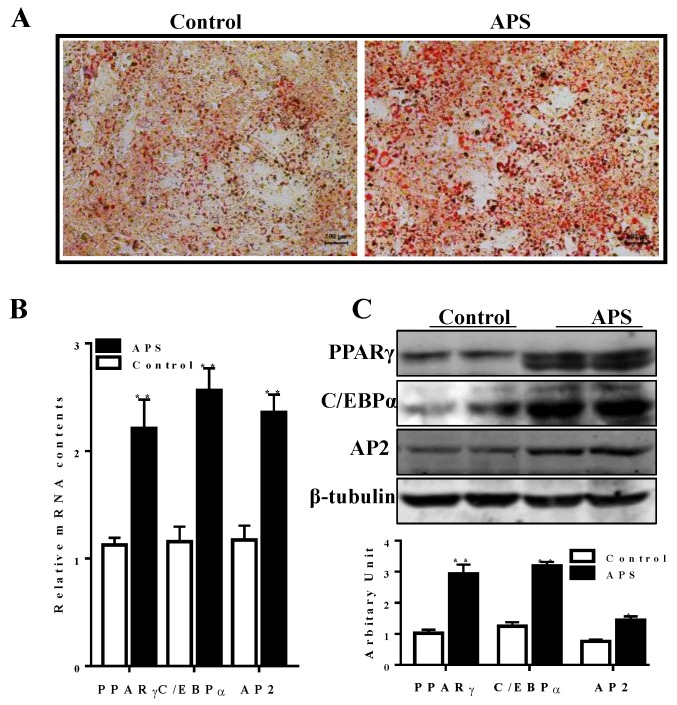
APS enhanced adipogenesis in 3T3-L1 pre-adipocytes. (**A**) Oil-red-O staining showed that more lipids were accumulated in the APS treated cells. (**B**) mRNA expression of adipogenic transcriptional factors, including proliferator-activated receptor γ (PPARγ), CCAAT/enhancer binding protein α (C/EBPα), and fatty acid binding protein (aP2). (**C**) Protein contents of PPARγ, C/EBPα, and aP2. n = 6, * *p* < 0.05, ** *p* < 0.01.

Oil-red-O staining demonstrated that the addition of APS increased lipid accumulation after 6 days of differentiation (Figure 2A). Compared with control cells, APS treatment enhanced mRNA expression of adipogenic transcriptional factors, including PPARγ, C/EBPα, and *fatty acid binding protein* (*aP2*) (*p* < 0.01, Figure 2B). Western blot analysis further confirmed the increased protein contents of PPARγ (*p* < 0.01), C/EBPα (*p* < 0.01) and aP2 (*p* < 0.05) (Figure 2C).

### 2.3. APS Stimulated Glucose Uptake and Increased the Expression of Glut4 

**Figure 3:**
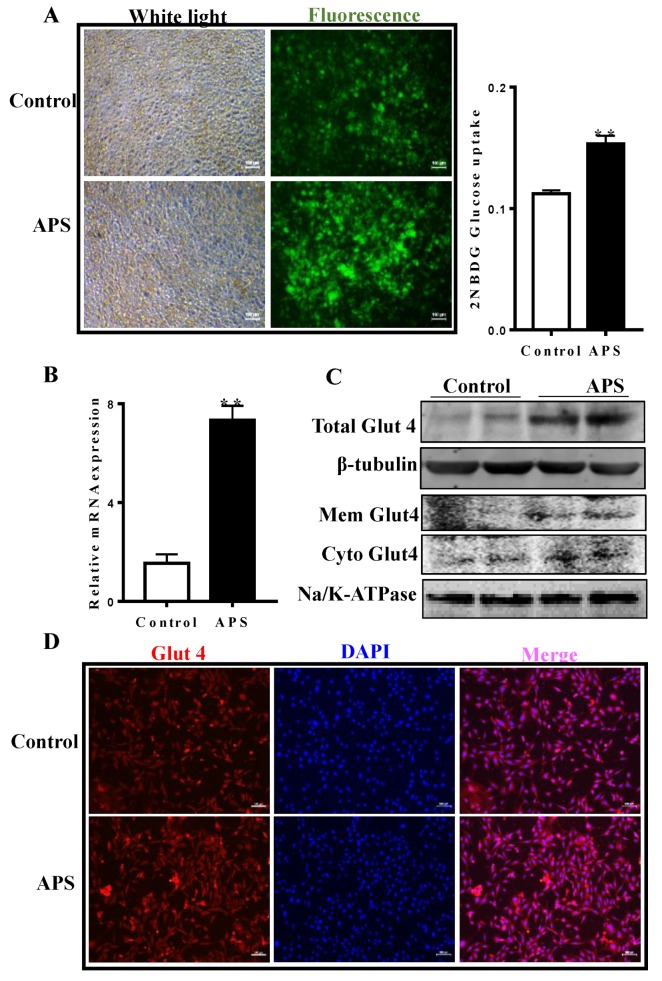
APS stimulated glucose uptake and increased the expression of glucose transporter 4 (Glut4) in adipocytes. (**A**) 2-(*N*-(7-Nitrobenz-2-oxa-1,3-diazol-4-yl)Amino)-2-Deoxyglucose (2-NBDG) uptake assay, data show that APS promoted glucose uptake in fully differentiated adipocytes. (**B**) mRNA in differentiated adipocytes treated with or without APS. (**C**) Total, membrane and cytoplasmic Glut4 content in different groups. (**D**) Immunocytochemical staining of Glut4. n = 6, ** *p* < 0.01.

A 2-(*N*-(7-Nitrobenz-2-oxa-1,3-diazol-4-yl)Amino)-2-Deoxyglucose (2-NBDG) assay was employed to monitor the effects of APS on glucose uptake in fully differentiated adipocytes. As shown in Figure 3A, the retained 2’NBDG-fluorescence in cells was increased by APS treatment compared to that of the control cells, and this was supported by quantitative data obtained from a spectrophotometer. qRT-PCR data suggested that *Glut4* mRNA content was increased by 7.5 times during adipocyte differentiation compared with control cells (*p* < 0.01, Figure 3B). As expected, APS addition increased the Glut4 protein level by 5.8 fold as compared with control cells (Figure 3C). Moreover, both the membrane and cytosol Glut4 contents were elevated in the APS treated cells (Figure 3C). Consistently, immunocytochemical staining further confirmed the effects of APS on cellular Glut4 content (Figure 3D). 

### 2.4. Effect of APS Treatment on Insulin Sensitivity and AMPK Activity

**Figure 4:**
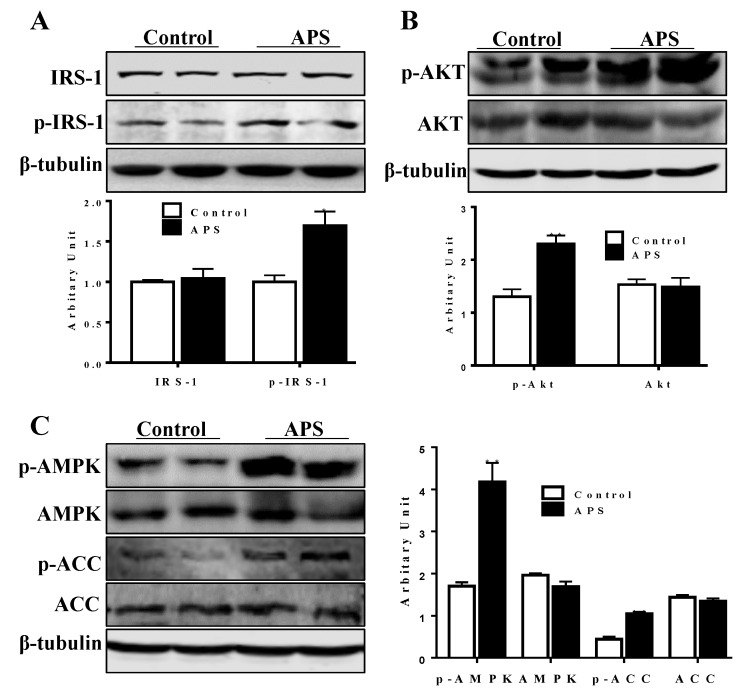
Effect of APS treatment on insulin receptor substrate protein 1 (IRS1), Akt, and AMP-activated protein kinase (AMPK) activity. (**A**) IRS1 and phosphorylated IRS1 (p-IRS1) protein contents. (**B**) Akt and phosphorylated Akt (p-Akt) protein contents. (**C**) Protein contents of AMPK, phosphorylated AMPK (p-AMPK), CoA carboxylase (ACC) and phosphorylated ACC (p-ACC). n = 6, * *p* < 0.05 and ** *p* < 0.01.

As shown in Figure 4A, compared with control cells, APS treatment effectively enhanced tyrosine phosphorylation of insulin receptor substrate protein 1 (IRS1) (*p* < 0.05) without alteration in total IRS1 protein content. Considering that Akt phosphorylation (p-Akt) is essential for the insulin signaling pathway, the effect of APS on Akt activation was investigated. Data suggested that cells treated with APS clearly exhibited an elevated p-Akt content (*p* < 0.01, Figure 4B). Moreover, phosphorylated AMPK content was increased in the APS treated cells (*p* < 0.01, Figure 4C). Acetyl-CoA carboxylase (ACC) is the direct substrate of AMPK, the phosphorylated ACC (p-ACC) indicated the activation of AMPK. Here, p-ACC content was higher in the APS treated cells, which further confirmed the activation of AMPK by APS.

## 3. Discussion

The loss of insulin sensitivity in target tissues, declining insulin production, and eventual pancreatic β-cell failure are the major features of T2DM. Thus, identifying the molecular basis of improving insulin sensitivity is a plausible therapeutic approach for T2DM and related metabolic diseases. Medicinal plants have been used empirically in antidiabetic and antihyperlipidemic remedies, and recently, many studies have focused on finding natural components that can be used as insulin sensitizers with slight or no side effects [16]. *Astragalus* polysaccharide has been studied in depth and is widely used in clinical settings. Previous studies demonstrate that APS can effectively reduce the blood sugar and the serum triglyceride, carbohydrate, and low-density lipoprotein content of type 2 diabetic rats, while increasing high density lipoprotein levels [11]. 

Cell exposure to cytotoxic substrates causes decreased cell viability or cellular death through necrosis or apoptosis [17]. A commonly used method for determining cytotoxicity is to measure the activity of cytoplasmic enzymes released by damaged cells. Addition of APS at certain concentrations did not induce cellular cytotoxicity in 3T3-L1 preadipocytes, suggesting its potential safety and therapeutic application in clinics. 

Adipogenesis involves cell proliferation and differentiation, and both processes are tightly regulated. Just 0.1 μg/mL APS sufficiently enhanced 3T3-L1 preadipocytes proliferation, which was further confirmed by the CCK8 assay, and activated AMPK. Since AMPK activation inhibits cell proliferation, the observed proliferation by APS might be from other unknown mechanisms that counteracted the inhibitory effects of AMPK. The PCNA protein is a standard marker of proliferation that is commonly used to assess the growth fraction of a cell population [18]. PCNA is involved in DNA replication, chromatin remodeling, DNA repair, sister-chromatid cohesion, and the cell cycle [19], and our data suggested that APS exerted proliferative effects through regulation of PCNA expression; the underlying mechanism of this needs to be further explored. The p38 mitogen-activated protein kinase (MAPK) plays an important role in mammalian cell proliferation. Interestingly, our data suggested that cells treated with APS exhibited a higher p38 activity, in accordance with previous reports showing that APS can activate p38 MAPK in mice hepatocytes [20]. 

Adipogenesis is regulated by multiple transcription factors, coregulators, and miRNAs [21]. It is well established that PPARγ and C/EBPα are recognized as master regulators of adipogenesis and act in concert to orchestrate the differentiation of preadipocytes into adipocytes [22]. The elevated PPARγ and C/EBPα contents suggested that effects of APS on adipocyte differentiation might be mediated through the up-regulation of adipogenic transcription factors, in accordance with previous results that showed that APS attenuates Tumor Necrosis Factor α (TNF-α) induced insulin resistance via activation of PPARγ in 3T3-L1 adipocytes [23]. aP2 is extensively used as a marker for differentiated adipocytes, and plays an important role in the intracellular fatty acid transport and metabolism [24]; an increased aP2 expression further confirmed the enhanced adipogenesis by APS. In addition to its function as an energy reservoir, white adipose tissue is an important endocrine and secretory organ. Adipose tissue actively communicates by sending and receiving different types of signals to perform normal physiological functions [25]. Therefore, the enhanced adipocytes differentiation by APS might alter its physiological functions, and the details and mechanisms need to be further explored in an animal model. 

Adipose tissue is one of the primary targets of insulin-stimulated glucose uptake [26]. The induction of adipogenesis by APS suggested its potential for enhancing insulin sensitivity and glucose uptake by adipocytes. Glucose uptake is dependent on glucose transporters, and Glut4 is the major transporter in adipocytes [27]. Our data suggested that during adipocyte differentiation, APS increased both the total and membrane fraction of Glut4 contents. Considering that insulin resistance in adipocytes primarily arises from depletion of Glut4 [14], up-regulation of Glut4 by APS contributed to improving insulin sensitivity in the 3T3-L1 adipocytes. In addition to adipocytes, a previous study showed that APS can alleviate glucose toxicity by increasing liver glycogen synthesis and skeletal muscle glucose translocation in the T2DM rat model [28]. Moreover, APS attenuates insulin resistance in T2DM rats via regulation of liver microRNA-203a-3p [13]. Collectively, these data suggest that APS could be a potential insulin sensitizer for the treatment of type 2 diabetes.

Glucose uptake requires the translocation of Glut4 proteins from the cytosol to the plasma membrane, and insulin signaling pathways play an important role in this process [29]. The binding of insulin to its receptor leads to the phosphorylation of tyrosine residues in IRS1, and then triggers the phosphorylation of PI3K and Akt, which further promotes Glut4 translocation and glucose uptake [30]. Here, APS increased phosphorylation of both tyrosine in IRS1 and Akt, suggesting that APS stimulates glucose uptake by activating the insulin-Akt signaling pathway. Adiponectin is an adipocyte-derived cytokine with insulin-sensitizing properties. A previous study shows that adiponectin sensitizes insulin signaling by reducing p70 S6 kinase-mediated serine phosphorylation, leading to enhanced IRS1 and Akt phosphorylation [31]. Thus, the enhanced adipogenesis by APS in the current study might have produced higher adiponectin, and contributed to the elevated phosphorylation of both IRS1 and Akt. 

AMPK belongs to a family of serine/threonine kinases and is a fuel sensor monitoring the AMP/ATP ratio. Various extracts or chemicals from medicinal herbs improve glucose uptake via AMPK activation [32]. In the present study, APS dramatically activated AMPK, in line with a previous study showing that APS activates AMPK in high-glucose treated C2C12 cells [33]. Although activation of AMPK by its activators is negatively associated with adipogenesis, other unknown positive effects exerted by APS might counteract its inhibitory effect, leading to the enhanced adipogenesis. A previous study demonstrates that AMPK regulates Glut4 transcription through the Histone deacetylase 5 (HDAC5) transcriptional repressor [34]. Moreover, activation of AMPK promotes Glut4 translocation and glucose transportation activity in 3T3-L1 adipocytes [35]. Thus, activation of AMPK might be a key contributor for APS induced insulin sensitivity in adipocytes. 

## 4. Materials and Methods 

### 4.1. Cell Culture

Mouse 3T3-L1 preadipocytes were purchased from American Type Culture Collection (ATCC, Manassas, VA, USA) and were grown in Dulbecco’s modified Eagle’s medium (DMEM) supplemented with 10% fetal bovine serum (FBS) and 1% antibiotics at 37 °C. After cells reached confluence, adipocyte differentiation was induced with or without APS in a mixture containing 1 μg/mL isobutyl-methylxanthine, 1 μM dexamethasone, and 1 μg/mL insulin for 2 days. Then, medium was changed to DMEM containing insulin (1 μg/mL) only with or without APS for 2 days and repeated once. APS (98% purity) purchased from YuanYe Biotechnology Co., Ltd. (Shanghai, China) was dissolved in DMEM medium at 100 mg/mL and diluted with the culture medium to obtain the needed sample concentrations before addition to cells. APS was used at 0.1 μg/mL in most of experiment unless otherwise noted.

### 4.2. Lactate Dehydrogenase (LDH) Assay

LDH activity on the supernatant was determined using an LDH assay kit according to the manufacturer’s instructions (CK28, Dojindo China Co., Ltd., Shanghai, China). Briefly, cells grown on a 96-well plate were treated with different concentration of APS (0, 0.1, 0.5, 2.5, 10 μg/mL) for 24 h. Then, 100 μL of the working solution was added to each well, and the plates was incubated at room temperature by protecting it from light for 30 mins. After that, 50 μL of the stop solution was added to each well, and the OD values at 490 nm were obtained in a spectrophotometer (Synergy H1 Multi-Mode Reader, Winooski, VT, USA). The cytotoxicity was calculated based on the provided equation. 

### 4.3. CCK-8 Assays

Cell proliferation was determined by CCK-8 (Dojindo, Osaka, Japan). The 3T3-L1 preadipocytes were seeded at 1 × 10^3^ per well in a 96-well plate and cultured in DMEM. When cells were attached to the bottom (about 6 h), different concentrations of APS (0, 0.1, 0.5, 2.5, 10 μg/mL) were added to the medium. After 24 h, 10 μL of CCK-8 reagent was added to the medium, and cells were incubated for 2 h. Finally, the absorbance at 450 nm was measured using a Microplate Reader (Synergy H1 Multi-Mode Reader, Winooski, VT, USA).

### 4.4. EdU Staining

Cell proliferation was assessed by a Cell-Light EdU DNA cell proliferation kit (C10310-1, RiboBio, Guangzhou, China) according to the manufacturer’s instructions. Briefly, 24 h after AICAR, Compound C, or APS treatment (0, 0.1, 0.5, 2.5, 10 μg/mL), 3T3-L1 preadipocytes were exposed to 50 μM EdU and were cultured for an additional 2 h. Next, EdU medium mixture was discarded, cells were fixed in 4% paraformaldehyde for 30 min and permeabilized with 0.5% Triton X-100 for 10 min. Subsequently, 1 × Apollo reaction cocktail (RiboBio) was added and incubated for 30 min while protecting it from light, and then the cells were stained with Hoechst 33342. Finally, EdU-stained cells were visualized under a DMi8 fluorescence microscope (Leica, Wetzlar, Germany).

### 4.5. Oil-Red-O Staining

3T3-L1 pre-adipocytes had adipogenesis induced with or without APS for 6 days (0.1 μg/mL). Differentiated adipocytes were washed with phosphate buffered saline (PBS) and fixed in 10% formaldehyde for 10 min at room temperature. Then, cells were rinsed three times with PBS and stained with 0.2% (*w*/*v*) Oil-Red O (Sigma Chemical Co., Saint Louis, MO, USA) for 20 min. After that, the plates were rinsed three times with distilled water, and images were taken at a 100× magnification under a DMi8 microscope (Leica, Wetzlar, Germany).

### 4.6. Western Blotting 

Differentiated adipocytes were homogenized and subjected to SDS-PAGE and immunoblotting analyses as previously described with the use of an Odyssey Infrared Imaging System (LI-COR Biosciences, Lincoln, NE, USA) [36]. Band density was normalized according to the β-tubulin content. Antibody details were as follows: PPARγ (sc-7196), C/EBPα (sc-9314), and aP2 (sc-18661) were purchased from Santa Cruz Biotechnology, Inc. (Santa Cruz, CA, USA). Akt (no. 9272), p-Akt (no. 2146), AMPK (no. 2532), phosphorylated AMPK (p-AMPK) (no. 2535), ACC (no. 3662), p-ACC (no. 3661), Glut4 (no. 2213), and β-tubulin (no. 2146) were from Cell Signaling (Danvers, MA, USA). IRS1 (bs-0172R), phosphorylated IRS1 (p-IRS1) (bs-2736R), PCNA (bs-0754R), and Na/K-ATPase (bs-4255R) were from Biosynthesis Biotechnology Co., Ltd. (Beijing, China). Goat anti-mouse secondary antibody (926-68070) and anti-rabbit secondary antibody (926-32211) were from LI-COR Biosciences (Lincoln, NE, USA).

### 4.7. Real-Time Quantitative PCR (qRT-PCR)

Differentiated adipocytes were harvested and total RNA was extracted using Trizol reagent (Sigma, Saint Louis, MO, USA) followed by DNase I (#M0303s, New England Biolabs, Ipswich, England) digestion. Concentration and the integrity of RNA samples were determined by both NanoDrop ND-2000 instrument (Nanodrop Instruments, Wilmington, DE, USA) and electrophoresis with 2% agarose gel. A total of 1 μg RNA was used for cDNA synthesis using a reverse transcription kit (TAKARA Co., Ltd., Dalian, China), with qRT-PCR performed using the CFX qRT-PCR detection system (Bio-Rad, Hercules, CA, USA) and a SYBR Green RT-PCR kit (TAKARA Co., Ltd., Dalian, China). Relative mRNA content was normalized to the *18S rRNA* gene. Primer sets used are listed in Table 1.

### 4.8. Plasma Membrane and Cytosol Fractionation

The membrane and cytosol fraction isolation were performed based on previously described methods [37]. Briefly, 3T3-L1 adipocytes treated with or without 0.1 μg/mL APS for 6 days were harvested. The cells were disrupted with RIPA lysis buffer and kept on ice for 20 min. Then, the lysates were centrifuged at 13,000× *g* for 5 min at 4 °C to obtain the supernatant. Subsequently, the supernatant was ultra-centrifuged at 77,000× *g* for 1 h at 4 °C, and both the precipitate (plasma membrane fraction) and supernatant (cytosol fraction) were collected for Glut4 analysis as Section 4.6 described. 

### 4.9. Glucose Uptake Assay

Glucose uptake was evaluated by the incorporation of 2-NBDG (Life Technologies, Eugene, Oregon, OR, USA, Cat: N13195), a d-glucose fluorescent derivative. Briefly, differentiated adipocytes treated with or without APS were serum-starved in DMEM for 12 h. Then, cells were washed 3 times with cold PBS (pH 7.4) and 100 μM 2-NBDG was added to the cells and co-incubated for 15 min at 37 °C. The 2-NBDG uptake reaction was stopped by removing the incubation medium and washing the cells twice with cold PBS. Finally, pictures were taken under DMi8 fluorescence microscope (Leica, Germany), and fluorescence was measured in a spectrophotometer (Synergy H1 Multi-Mode Reader, Winooski, VT, USA). 

### 4.10. Immunocytochemical Staining

Cells grown on coverslips were fixed in cold methanol for 10 min and permeabilized with PBS containing 0.25% Trition X-100 for 10 min. After blocking 1 h with 1% Bovine serum albumin (BSA), cells were incubated with Glut4 antibody (1：200) (Cell Signaling, Danvers, MA, USA, no. 2213) at 4 °C overnight. After washing 3 times with Phosphate-Buffered Saline with Tween 20 (PBST), fluorescent secondary antibody (1:1000) was then added and incubated for 1 h. Fluorescence was examined by using DMi8 microscope (Leica, Wetzlar, Germany).

### 4.11. Statistical Analysis

Statistical analysis was performed using the GraphPad Prism 6 software package (Monrovia, CA, USA), and data were expressed as mean ± SEM. For most of the assays, data were analyzed using Student’s *t* test. For LDH assay, CCK8 assay, and EdU staining, normality of data and homogeneity of variance were tested using the Shapiro-Wilk and the Brown-Forsythe test. Normally distributed data were analyzed using analysis of variance (ANOVA), and Duncan’s multiple-range test was used for *post hoc*. *p* < 0.05 was considered as statistical significance for all data. 

## 5. Conclusions

APS effectively promoted 3T3-L1 preadipocytes proliferation and terminal differentiation. Moreover, APS up-regulated Glut4 expression and increased glucose uptake, which was associated with activation of AMPK. Collectively, APS could be used as an insulin sensitizer, which may exert effects at multiple sites.

## Figures and Tables

**Table 1:** Primer sequences for real-time PCR.

Name	Sequence (5′–3′)	Accession Number
PCNA	GAACCTCACCAGCATGTCCA TGGGATTCCAAGTTGCTCCA	NM_011045.2
PPARγ	ACCTCTGCTGGGGATCTGAA ATCACGGAGAGGTCCACAGA	NM_001127330.2
C/EBPα	CCCTTGCTTTTTGCACCTCC TGCCCCCATTCTCCATGAAC	NM_001287514.1
AP2	GGATTTGGTCACCATCCGGT TTCCATCCCACTTCTGCACC	NM_024406.3
Glut4	CTAGGCATCAATGCTGTTTTCTA CGAGACCAACGTGAAGACCGTATT	AB008453.1
18S	GTAACCCGTTGAACCCCATT CCATCCAATCGGTAGTAGCG	M35283.1

## Supplementary Materials

The supplementary materials are available online.
