# Supplementary material for: Astragalus Polysaccharide Improves Insulin Sensitivity via AMPK Activation in 3T3-L1 Adipocytes
Source: Molecules. 2018 Oct 21;23(10):2711. doi: 10.3390/molecules23102711 (PMC6222405; doi:10.3390/molecules23102711)
Supplement: Supplementary file 1 [file molecules-23-02711-s001.pdf]

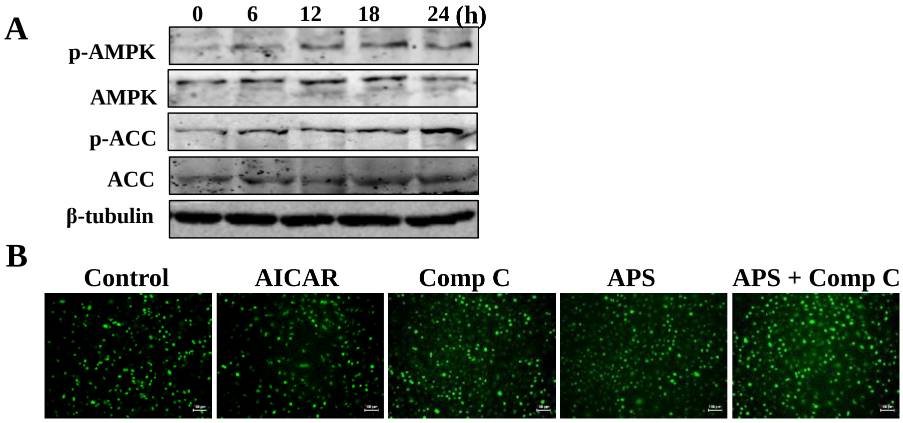

Supplementary Figure: APS activates AMPK and promotes 3T3-L1 proliferation. (A) AMPK activity at different time points (0, 6, 12, 18, 24 h). (B) EdU staining after cells were treated with AICAR, Compound C, APS, and APS + Compound C. Scar bar stands for 100 μm.
